# IMG/M 4 version of the integrated metagenome comparative analysis system

**DOI:** 10.1093/nar/gkt919

**Published:** 2013-10-16

**Authors:** Victor M. Markowitz, I-Min A. Chen, Ken Chu, Ernest Szeto, Krishna Palaniappan, Manoj Pillay, Anna Ratner, Jinghua Huang, Ioanna Pagani, Susannah Tringe, Marcel Huntemann, Konstantinos Billis, Neha Varghese, Kristin Tennessen, Konstantinos Mavromatis, Amrita Pati, Natalia N. Ivanova, Nikos C. Kyrpides

**Affiliations:** ^1^Biological Data Management and Technology Center, Computational Research Division Lawrence Berkeley National Laboratory, 1 Cyclotron Road, Berkeley, 94720 USA and ^2^Microbial Genome and Metagenome Program, Department of Energy Joint Genome Institute, 2800 Mitchell Drive, Walnut Creek, CA, 94598 USA

## Abstract

IMG/M (http://img.jgi.doe.gov/m) provides support for comparative analysis of microbial community aggregate genomes (metagenomes) in the context of a comprehensive set of reference genomes from all three domains of life, as well as plasmids, viruses and genome fragments. IMG/M’s data content and analytical tools have expanded continuously since its first version was released in 2007. Since the last report published in the 2012 NAR Database Issue, IMG/M’s database architecture, annotation and data integration pipelines and analysis tools have been extended to copewith the rapid growth in the number and size of metagenome data sets handled by the system. IMG/M data marts provide support for the analysis of publicly available genomes, expert review of metagenome annotations (IMG/M ER: http://img.jgi.doe.gov/mer) and Human Microbiome Project (HMP)-specific metagenome samples (IMG/M HMP: http://img.jgi.doe.gov/imgm_hmp).

## DATA SOURCES AND PROCESSING

Metagenome analysis involves examining the phylogenetic composition and functional or metabolic potential of microbiomes in the context of a large set of reference genomes. Consequently, IMG/M consists of samples of microbial community (microbiome) aggregate genomes (metagenomes) integrated with IMG’s comprehensive set of genomes from all three domains of life: plasmids, viruses and genome fragments (1). The IMG ER Submission system (http://img.jgi.doe.gov/submit) is used for managing the processing and integration of metagenome data sets. First, the metagenome data sets submitted for inclusion into IMG/M ER are associated in GOLD (2) with metadata attributes following the Genome Standards Consortium guidelines (3) and classified using a multi-tiered system. Thus, all metagenome data sets are organized in three main *ecosystem classes*, *environmental*, *host associated* and *engineered*, and then further divided in subclasses characterized by *ecosystem categories* (e.g. aquatic, terrestrial, air for environmental metagenomes), *ecosystem type* (e.g. freshwater, marine), *ecosystem subtype* (e.g. groundwater, drinking water) and *specific ecosystem* (e.g. cave water, coalbed water). The GOLD metagenome classification and metadata attributes are included into IMG/M where they are essential for selecting metagenome data sets for comparative analysis.

For both genomes and metagenomes, IMG/M records their primary sequence information, their organization in scaffolds and/or contigs, as well as computationally predicted protein-coding sequences, some RNA-coding genes and protein product names. Metagenome sequences are prepared for annotation by (i) removing commonly occurring discrepancies in the input sequence files, such as duplicate sequence identifiers, and by replacing ambiguous nucleotides by Ns, while sequences with characters not occurring in {A,C,G,T,N} are not considered further; (ii) trimming sequences to remove low-quality regions and trailing ‘N’s; (iii) masking low-complexity regions identified using DUST (4); (iv) retaining only one copy when two or more sequences are >95% identical (dereplication).

Metagenome gene prediction starts with the detection of CRISPR elements using CRT (5) and PILERCR (6). Predictions from both methods are concatenated, and in case of overlapping elements, the shorter one is removed. Identification of tRNAs is performed using tRNAScan-SE-1.23 (7). A metagenome is a potential mixture of the three domains of life, so the program is run three times, one for each domain (*Bacteria*, *Archaea, Eukaryota*), with custom parameters for each. The best scoring predictions are then selected. Since the program cannot detect fragmented tRNAs at the ends of sequences, sequences are compared with a database of nt sequences of tRNAs identified in all isolate genomes (For sequences longer than 300 bp, only the first 150 bp and the last 150 bp are matched). Hits with high similarity (at least 85% identity and a minimum alignment length of 40) are kept. Protein-coding genes are identified using four *ab initio* gene calling tools: GeneMark (8), Metagene (9), Prodigal (10) and FragGeneScan (11). A majority rule-based decision system is followed to select protein-coding genes, which are then consolidated in terms of resolution of overlaps. In the event of an overlap between a protein-coding gene and an RNA gene or CRISPR element, the RNA gene or CRISPR element is retained, while allowing small 3′-3′ overlaps between protein coding and RNA genes.

Metagenome protein-coding genes are compared with protein families and the proteome of selected publicly available ‘core’ genomes, with product names assigned based on the results of these comparisons. First, protein sequences are compared with COG (12) using RPS-BLAST and Pfam-A (13) using HMMER 3. Metagenome protein-coding genes are associated with KEGG Orthology terms (14), EC numbers and phylogeny using USEARCH (15) similarity searches against a reference database consisting of all nonredundant protein sequences from the public genomes available in IMG and the KEGG database (14). The integration of new metagenomes into IMG/M involves computing protein sequence similarities between their genes and genes of all reference genomes in the system and assigning protein product names to the genes of the new metagenomes based on their associated COGs or Pfams.

## DATA CONTENT

### Metagenome and reference genome data

The number of metagenome data sets in IMG/M has increased substantially since the last published report on IMG/M (16). The current version of IMG/M (as of September 10, 2013) contains 3328 metagenome data sets from 460 metagenome studies, with >19.5 billion protein coding genes [IMG/M contained 870 metagenome data sets from 227 studies with 163 million protein coding genes in October 2011 at the time the last published report on the system was prepared (16)]. About 2093 metagenome data sets are publicly available to all users via the IMG/M datamart (http://img.jgi.doe.gov/m). These data sets are organized using a habitat-based classification (17) and include 80 engineered, 1144 environmental and 869 host-associated metagenomes (Table 1).

**Table 1.** Habitat-based metagenomic classification in IMG/M

Engineered	80	Environmental	1144	Host-associated	869
Bioremediation	17	Air	2	Arthropoda	53
Biotransformation	9	Aquatic	779	Birds	6
Solid waste	25	Terrestrial	363	Human	753
Wastewater	29			Mammals	18
				Microbial	1
				Mollusca	9
				Plants	27
				Porifera	2

Metagenome data sets that have not been yet published (also known as ‘private’) are password protected and available only to the scientists who study (‘own’) them through the IMG/M ER (‘Expert Review’) datamart (http://img.jgi.doe.gov/mer). Private metagenome data sets are usually publicly released 12 months after they become available in IMG/M.

Metagenome data sets are integrated with IMG’s set of publicly available reference genomes. The current version of IMG (as of September 10, 2013) contains >13 300 reference genomes consisting of 8761 bacterial, archaeal and eukaryotic genomes, as well as 2848 viral genomes, 1198 plasmids that did not come from a specific microbial genome sequencing project and 581 genome fragments, with ∼33 million protein-coding genes. Genomes generated as part of the Microbial Dark Matter project (18), which aims to use single cell genomics to expand the Genomic Encyclopedia of Bacteria and Archaea (19) by targeting single cell representatives of uncultured candidate phyla are of particular importance to the breadth of the reference set of genomes involved in metagenome analysis. The number of single-cell genomes available in IMG has increased from only 21 available in August 2011 to >1340 in September 2013.

A Human Microbiome Project (HMP) IMG/M data mart (http://img.jgi.doe.gov/imgm_hmp) contains 748 metagenome data sets generated by sequencing samples collected from various body sites (airways, gastrointestinal, oral, skin, urogenital), as part of the HMP initiative (20). In addition to the HMP-specific data sets, IMG/M contains >130 additional human-associated metagenome samples that are part of various studies. Metagenome and genome data sets in IMG/M-HMP are grouped both by body site category and by taxonomy, while metagenome data sets are also grouped according to the primary body site and human subjects sampled. HMP-specific analysis ‘workflows’ are discussed in (21).

### Metatranscriptome data

**Figure 1.**
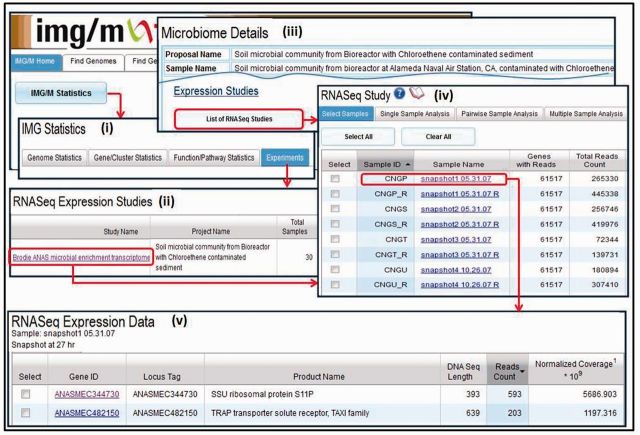
RNA-Seq data organization. (i) Metatranscriptomic (RNA-Seq) data sets can be accessed from ‘IMG Statistics’ on IMG/M’s front page, following the experiments link available on the ‘IMG Statistics’ page. (ii) An RNA-Seq study is associated with a metagenome project (assembly onto which the RNA-Seq reads have been mapped) and a number of samples. (iii) RNA-Seq studies associated with a metagenome project can be accessed from its ‘Microbiome Details’, with each study associated with (iv) a list of RNA-Seq experiments (samples). Individual samples can be selected for further analysis, such as (v) examining its expressed genes as a list.

The first metatranscriptomic (RNA-Seq) data sets were included into IMG/M in 2012, with IMG/M currently (as of August 2013) containing >160 samples across 16 RNASeq studies. Metatranscriptome studies are sequencing projects that have one or more samples associated with different conditions. A metatranscriptome study may be part of a systematic study with a metagenome counterpart or it may be an isolated study involving just the metatranscriptome. Samples undergo RNASeq sequencing analysis, where reads are mapped to the reference isolate or metagenome(s) described in the study, and the expressed genes in each sample are recorded with their observed reads count, mean, median and strand. Additionally, reads from every metatranscriptome are assembled *de novo*, and the assembly is annotated with the regular metagenome pipeline. Transcripts are then mapped onto this assembly. RNA reads are mapped to reference genomes/assemblies using Bowtie2 (22). The scope of mapping is determined by the type of cDNA sample (sscDNA/dscDNA) and the directionality of the libraries, whereby reads may map to a single strand or both strands of the reference sequence. Expression levels are normalized by computing reads per kilobase per million quantile or affine transformations. For genomes involved in RNASeq studies, the experiments/samples are recorded in IMG together with experimental conditions, and the read counts are organized per expressed gene, as illustrated in Figure 1.

## DATA ANALYSIS

Browsers and search tools allow finding and selecting metagenome samples, genomes, genes and functions of interest, which can then be examined individually or analyzed in a comparative context. The composition of analysis operations is facilitated by (meta)genome, scaffold, gene and function ‘carts’ that handle lists of genomes, scaffolds, genes and functions, respectively. The phylogenetic composition of a metagenome sample is provided by computing the distribution of the best BLAST hits of the protein-coding genes in the sample against the reference genomes.

Function-based comparison of metagenome samples and genomes is provided by analysis tools that allow examining the relative abundance of protein families (COGs, Pfams, TIGRfams), functional families (enzymes) or functional categories (COG Pathway, KEGG Pathway, KEGG Pathway Category, Pfam Category) across metagenome samples and genomes. These comparisons take into account the stochastic nature of metagenome data sets and test whether differences in abundance can be ascribed to chance variation or not.

**Figure 2.**
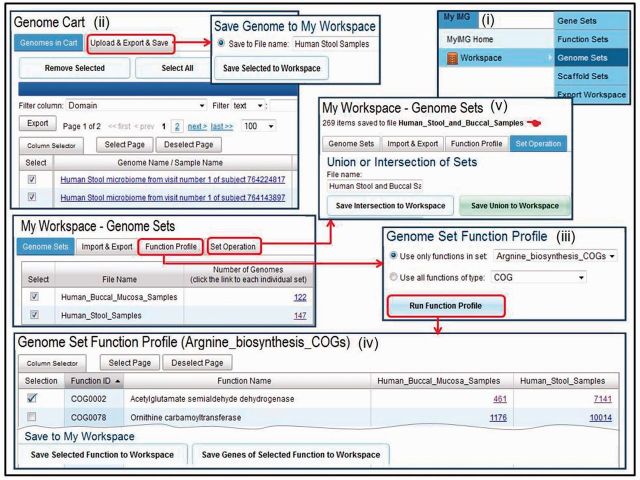
Using workspace tools to analyze metagenome samples. (i) ‘Workspace’ is part of the ‘MyIMG’ toolkit and consists of user-specified ‘Gene Sets’, ‘Genome Sets’, ‘Function Sets’ and ‘Scaffold Sets’, which can be created by (ii) transferring objects from the corresponding ‘Carts’ or from the lists of objects retrieved by IMG analysis tools, such as similarity searches or ‘Phylogenetic Distribution of Genes’. (iii) ‘Workspace’ supports profile operations between different sets of objects, with (iv) the results used to define new sets of genes and functions. (v) ‘Workspace’ operations on sets of objects include union, intersection and subtraction, with the results saved as new sets of objects.

Metagenome analysis tools have been discussed in previous reports on IMG/M. The new metagenome analysis tools developed since the last published report on IMG/M (16) are briefly reviewed below. These tools focus on handling substantially larger metagenome data sets, are available only to registered users as part of the ‘My IMG’ toolkit, as illustrated in Figure 2(i), and support specifying, managing and analyzing persistent sets of genes, functions, genomes or metagenome samples and scaffolds.

Sets of genes, functions, genomes/metagenome samples and scaffolds can be specified using the ‘Gene Cart’, ‘Function Cart’, ‘Genome Cart’ and ‘Scaffold Cart’, respectively. For example, two sets of metagenome samples are first specified using the ‘Genome Cart’ and then saved as named files into a user-specific ‘Workspace’, as illustrated in Figure 2(ii).

Sets of genes, functions, genomes/metagenome samples and scaffolds can be exported from (downloaded) or imported (uploaded) into IMG’s ‘Workspace’, and can be involved in set-based ‘Function Profile’, as illustrated in Figure 2(iii) where two sets of metagenome samples are compared in terms of a predefined set of (*Arginine biosynthesis*) COG functions. The ‘Function Profile’ result shown in Figure 2(iv) displays the number of genes associated with a specific function (COG) in the function set, across all the samples in the set of metagenome samples. The genes associated with a specific function can be used to specify a new set of genes in the user’s ‘Workspace’, as shown in the bottom part of Figure 2(iv). Set operations (intersection, union, difference) can be applied on sets of genes, functions, genomes and scaffolds, as illustrated in Figure 2(v) where union is applied on two sets of metagenome samples to create a new set of samples.

The workspace tools can be used for specifying metagenome or genome ‘bins**’** consisting of subsets of scaffolds. For example, single-cell genomes are typically screened for potential contamination, with scaffold sets used for separating ‘contaminated’ scaffolds from ‘clean’ scaffolds (http://img.jgi.doe.gov/w/doc/SingleCellDataDecontamination.pdf). For metagenomes, scaffold sets can be used for specifying genomes detected within/isolated from a microbial community. A scaffold set can be converted into a Fasta file using the ‘Workspace’ ‘Data Export’ tool and then resubmitted for data annotation and integration into IMG as a new data set.

**Figure 3.**
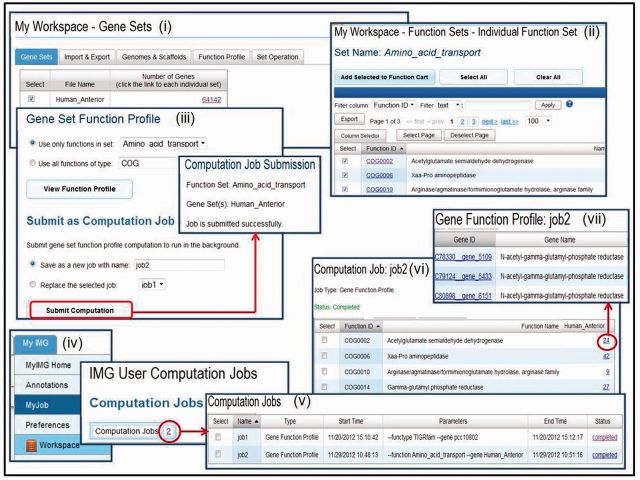
Background computations support analysis involving large sets of genes, functions and scaffolds, which can be specified using ‘Workspace’ (i) ‘Gene Sets’, (ii) ‘Function Sets’ and ‘Scaffold Sets’. Thus, (iii) a ‘Function Profile’ can be submitted as a background computation, whereby (iv) its status can be checked with ‘MyJob’. (v) For completed computations links are provided for accessing the analysis results, and (vii) associated details.

With the rapid growth of the number of genes in individual metagenome data sets, analysis operations may involve large sets (e.g. millions) of genes or a large number (e.g. hundreds) of scaffolds. Such operations require a long time (tens of minutes to hours) to complete, may time out in interactive mode, and therefore need to be executed off line as background computations. The mechanism for performing analysis operations as background computations is available as part of IMG’s ‘Workspace’ to IMG registered users. Background computations are supported for ‘Gene Function Profile’, ‘Scaffold Function Profile’ and ‘Scaffold Phylogenetic Distribution’ analyses, as illustrated in Figure 3.

For example, consider a function profile involving a gene set consisting of >64 000 human anterior microbiome genes, as illustrated in Figure 3(i), across a large number of COG and Pfam functions related to amino acid transport and metabolism, as illustrated in Figure 3(ii). After selecting the ‘Human_Anterior’ gene set in the ‘Gene Sets’ section of IMG’s ‘Workspace’, ‘Function Profile’ involving ‘Amino_acid_transport’ as a function set is submitted as a background computation, as illustrated in Figure 3(iii).

The status of a background computation is provided via the ‘MyJob’ section of the ‘MyIMG’ menu option, as illustrated in Figure 3(iv). When a computation (e.g. job 2) is ‘completed’, as illustrated in Figure 3(v), links are provided for accessing the analysis results, as illustrated in Figure 3(vi), and associated details, as illustrated in Figure 3(vii). The results of background computations are saved until users either explicitly delete the jobs using the ‘Delete’ option in the ‘Computation Jobs’ page, or override them with new jobs using the ‘Replace the selected job’ option.

## FUTURE PLANS

The current version of IMG/M (as of August 24, 2013) contains 3308 metagenome data sets from 460 metagenome studies. These data sets can be analyzed in the context of >13 000 bacterial, archaeal, eukaryotic and virus reference genomes. New metagenome data sets are continuously included into IMG/M from metagenome studies conducted at JGI and other institutes, while new isolate reference genomes are included from IMG on a regular basis. The number of metatranscriptomics data sets included into IMG/M is expected to grow rapidly in the next 2 years, with metaproteomics data sets also becoming available.

IMG’s maintenance involves continuously adjusting the underlying data management infrastructure to cope with the rapid increase in the number and size of the genome and metagenome data sets and to accommodate new data types. As we expect a steady growth in the number and size of metagenome data sets processed by and integrated into IMG/M, we continue to explore new data management techniques for organizing metagenome data sets and for providing support of effective metagenome data analysis.

## FUNDING

Director, Office of Science, Office of Biological and Environmental Research, Life Sciences Division, US Department of Energy under Contract No. [DE-AC02-05CH11231]. This research used resources of the National Energy Research Scientific Computing Center, which is supported by the Office of Science of the US Department of Energy under Contract No. [DE-AC02-05CH11231]. The IMG/M-HMP system is supported by the US National Institutes of Health Data Analysis and Coordination Center contract [U01-HG004866]. Funding for open access charge: University of California.

*Conflict of interest statement*. None declared.
